# Natural upper anterior teeth display an increasing proportion in mesio-distal direction

**DOI:** 10.4317/jced.56206

**Published:** 2019-10-01

**Authors:** Nutthapong Kantrong, Kritchaya Traiveat, Suchart Wongkhantee

**Affiliations:** 1 Department of Restorative Dentistry, Faculty of Dentistry, Khon Kaen University, Khon Kaen, Thailand; 2 Dental division, Srinagarind Hospital, Khon Kaen, Thailand

## Abstract

**Background:**

This study aimed to determine the esthetic proportion of six natural upper anterior teeth in northeastern Thai population as well as the level of esthetic satisfaction of distinct tooth proportions.

**Material and Methods:**

Front-view photographs were taken from participants living in the Northeastern Thailand (n=140) of the 18-35 years of age. Computerized program was used for measuring the size of each tooth. All subjects also scored the satisfaction level of different photographs portraying 4 proportions of anterior teeth (golden proportion, 70% RED, 80% RED, and an increasing proportion).

**Results:**

We found that proportion of lateral-to-central incisor and canine-to-lateral incisor were 0.72 and 0.80, respectively on both sides. The proportions increased in mesio-distal direction. Our reported ratios were statistically different (*P*<0.05) from the golden proportion, golden percentage, and 70% RED. However, the ratio of lateral-to-central incisor, but not the canine-to-lateral ratio, was significantly different (*P*<0.05) when compared to 80% RED. Esthetic satisfaction level of 4 tooth proportions among northeastern Thais was not statistically different (*P*=0.054).

**Conclusions:**

An increasing proportion of upper anterior teeth in the northeastern Thai subpopulation was found. No difference of esthetic satisfaction of 4 different tooth proportions among Thai laypersons warrants further study.

** Key words:**Esthetic proportion, Natural upper anterior teeth, Golden proportion, Increasing proportion, Esthetic satisfaction.

## Introduction

Smile is an essential feature of a personal, emotional expression that reflects an individual personality. An attractive smile consists of several components including an esthetic balance between lip feature, gum, and teeth (1-3). Proposed esthetic proportions i.e. golden proportion, golden percentage, Preston’s proportion, and Recurring Esthetic Dental proportion (RED) prevail for clinical considerations of anterior tooth restoration in which esthetic components are remarkably concerned. Esthetic proportions derived from the mathematical exposition are clinically utilized for predicting the width of each anterior tooth in order to reconstruct a natural anterior profile that portrays a harmonious tooth proportion (4-6).

Golden proportion, a known esthetic proportion clearly illustrated by nature, was introduced by Lombardi in 1973 for esthetic considerations in clinical dentistry (4) and further developed by Levin (5). In addition, it was first used for calculating the relationship of successive width of the anterior teeth and a corresponding smile (6,7). However, earlier studies have shown that the width of anterior natural teeth does not correspond to the golden proportion (8-13). Preston’s proportion and RED were thus introduced as alternates for the golden proportion, despite the difference of their reported values (6,10). The average width of maxillary lateral incisor was 66% of central incisor (10), while a Preston’s proportion calculated from maxillary canine-to-lateral incisor was shown to be 84% (6). Notably, Ward introduced RED in 2000 in which the proportion was derived from the ratio of incisal width between an anterior tooth and its juxtaposing tooth located mesially, as portrayed from the front view. RED is reproducible in one individual and the values derived from different persons might range from 66 to 78% (6). Although there are numerous esthetic proportions proposed to date, these proportions are not always clinically applicable to all population providing that ethnicity, as well as gender, reportedly plays a regulatory role in the determination of tooth shape and its appearance (14).

Our preliminary study to determine the natural width-to-height ratio of upper anterior teeth conducted in Khon Kaen, a central city of the northeastern region of Thailand, revealed that this ratio was not affected by gender (unpublished data). We found that a width-to-height ratio of the maxillary central incisor was 0.89, while the ratios calculated from maxillary lateral incisor and canine were 0.82-0.83 and 0.87-0.89, respectively. Our findings were consistent with previous studies concluding that ethnicity might be accountable for the variable features of natural tooth forms and esthetic proportions hence differing between the diverse studied population (14). Since our initial findings were derived from a limited number of study population, whether our preliminary results can be extrapolated to the majority of the Thais, particularly indigenous northeastern population, and in accordance with earlier reported ratios has become our research interest and therefore required further investigation.

It is noteworthy that a satisfaction of the patients on a tooth-associated esthetic profile contributes to a successful operative dental treatment. A clinical study in Canadian population indicated that the perception of a smile characteristics is affected by cultural differences (15). Interestingly, a clinical survey in laypersons to investigate their perspectives on the smile components revealed a restricted range of acceptance and esthetic preferences (15). In this study, we examined the esthetic proportions of natural upper anterior teeth in the indigenous, northeastern Thais as well as investigated their preference and satisfaction of varying smiles in which the 4 distinct proportions of upper anterior teeth were displayed. Ultimately, our findings might shed lights on how patients perceive towards esthetic elements related to dentistry and thus serve as a clinical tool, incorporated into a judgmental call of the dentists, for treatment planning in operative dentistry to surpass their expectation of treatment outcomes.

## Material and Methods

Our cross-sectional study aimed to determine the esthetic proportion and esthetic satisfaction of 6 upper anterior natural teeth in the Northeastern Thai population ranging between 18-35 years of age. The study proposal was reviewed by the Khon Kaen University Ethics Committee in human research and approved, based on the Declaration of Helsinki and the ICH Good Clinical Practices guidelines (Reference number HE592219). Five selected major geographical sites in the Northeast were chosen and data were collected from the major educational institution located in each area.

-Study population

A study was conducted in our select population (n=140) in which 28 persons per area from 5 major cities located in the northeastern Thailand were recruited into the study. All subjects participated in the study displayed a clinical, facial midline that was located on a dental midline in a maximum intercuspal position (MIP), fully-erupted upper anterior teeth, healthy gingiva without any tooth defect. Neither individuals who exhibited a notable deformity of the tooth surface such as enamel hypoplasia, cervical lesions nor any restoration in the anterior region was included. Subject who had received, was coincidentally undergoing an orthodontic treatment, or manifested a malocclusion i.e. crowding, spacing, openbite and crossbite was excluded from the study.

-Front-view photograph taking

Prior to data acquisition, plaque removal was done by using pumice powder in combination with rubber cup or gauze strip. All participants were then positioned at 30 cm-distance in front of the background wall. A photograph of the front view was taken 30 cm far from a subject as shown using tripods in which the setup location was marked using an adhesive tape to ensure an experimental replication. Participants were asked to bite at MIP and held a reference object, a metal sphere of a 1.5 cm diameter, at the area of mandibular incisors. A digital single-lens reflex camera (650D, Canon, Japan) with 100 mm macro lens (Canon, 100 mm 1:2.8 USM) and a ring flash (Canon Macro Ring Lite, MR-14EX) was setup at 1:2 magnification, ISO200, shutter speed of 1/200 F20 and placed on a tripods during the entire procedure.

**Figure 1:**
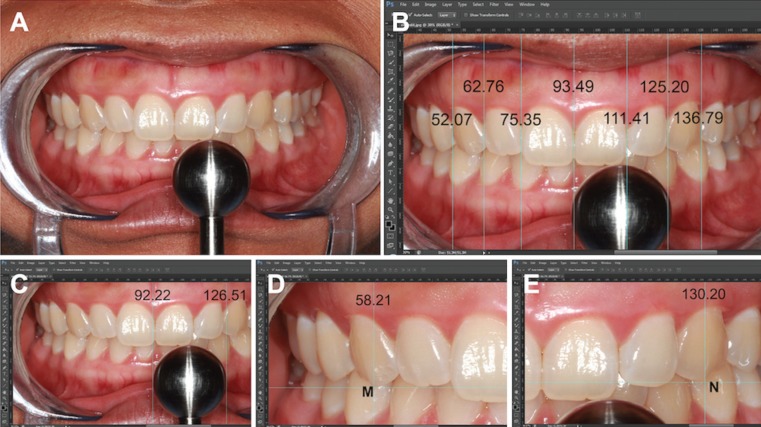
Image consisting of all upper anterior natural teeth was taken. (1A) A metal sphere of 1.5 cm diameter was incorporated in an image for a precise calculation of tooth width. (1B) Vertical lines passing through the contact areas were drawn using Adobe Photoshop program®. Each line was clearly located at the distal surface of each tooth when seen from the front view. Seven values of the distance were recorded for further analysis. (1C) A pictorial width of a metal sphere was used for calculating a tooth width. (1D and 1E) Canine tips were located (M and N) and used for calculating the distance between two canines (N-M).

In order to obtain standardized, comparable photographs, we positioned a critical intraoral landmark in the reproducible site during each capture i.e. interdental papilla between upper central incisors was laid at the center of the image frame. In addition, an incisal plane of upper central incisors was placed in parallel to the horizontal plane. Three photographs of similar components were taken before being transferred to a computer with Adobe Photoshop® (CS5 extended version 12.0.3 x32, Adobe System INC, San Lose, CA) installed for further analysis as seen in Figure 1A. Seven vertical lines were subsequently made manually by using the program to locate contact areas at both mesial and distal directions of each anterior tooth in which the horizontal distance of each line was generated automatically by the program as demonstrated in Figure 1B. In addition, two vertical lines located on the mesial and distal surfaces of the metal sphere were established as shown in Figure 1C. Additional vertical lines shown in Figure 1D and 1E passing the tip of upper canines from which the distance between canines can be estimated were also made. All vertical estimates were recorded in our spreadsheet for data analysis. All measurements were repeated for two more times in each image in order to achieve an approximate mean. To ensure the reproducibility and reliability of the measurement, each photograph was evaluated twice with a 1-week run-in period to obtain the average ± standard deviation derived from one individual participant.

-Calculating the esthetic ratio of upper anterior teeth

**Figure 2:**
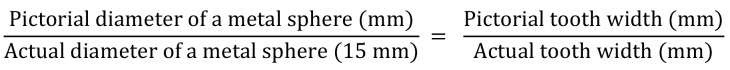
Formula.

After measuring a pictorial width of the teeth, the width of each tooth seen from a front view was calculated using a metal sphere as a reference object with known dimension. A mathematical exposition was performed by utilizing the following formula and recorded in our data sheet afterwards, (Fig. 2):

We next calculated the ratios of lateral-to-central incisor, ratio of canine-to-lateral incisor, golden percentage of the upper anterior teeth. These resultant ratios were further used for comparing with the golden proportion, 70% RED, and 80% RED.

-A survey of esthetic satisfaction of a smile portraying distinct tooth proportion

**Figure 3:**
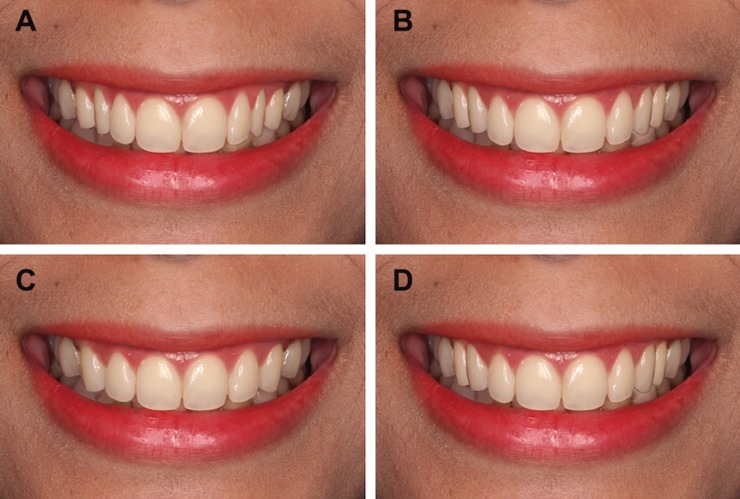
Four images portraying distinct tooth proportions were utilized in a clinical survey of esthetic satisfaction level of upper anterior natural teeth. (2A) Golden proportion. (2B) 70% RED. (2C) 80% RED. (2D) an increasing proportion.

Next, all participants were asked to score the level of esthetic satisfaction of different images portraying a smile displaying a distinct ratio between pairs of upper anterior teeth. An alteration of a prototypical smile was performed by using Adobe Photoshop® CS5 to illustrate the golden proportion (Fig. 3A), 70% RED (Fig. 3B), 80%RED (Fig. 3C), and an increasing proportion (Fig. 3D) of upper anterior teeth in a separated image. All 4 constructed photographs utilized the identical size of central incisor as a control. In order to construct a natural smile portraying an increasing proportion of upper anterior tooth, 70% RED and 80% RED were used to construct proportionate upper lateral incisor and canine respectively, thereby increasing the proportion from 0.7 to 0.8 from mesio-distal direction. Four modified photographs were subsequently presented to all subjects during which they were subject to scoring from 1 (least satisfactory) to 4 (most satisfactory).

-Data analysis

The average and standard deviation of the calculated ratios were derived from 3 independent measurements of the identical images taken from all subjects (n=140). One-sample t-test was performed to compare the tooth proportions derived from our study to the pre-existing ratios i.e. golden proportion, 70%RED, 80%RED, and an increasing proportion. Furthermore, an independent sample t-test was used to determine the difference between groups at the significance level of 0.05.

## Results

Total 140 subjects were recruited into the study: 55 males and 85 females with overall mean age of 22.22 ± 4.22 years. Two-dimensional tooth width of each anterior tooth was calculated using a metal sphere as a reference object and compared between genders. Consistent with previous report (16), the size of upper anterior teeth decreases in mesial-to-distal direction as shown in Table 1. In addition, our data in  Table 1 have indicated the difference of canine width between males and females with larger canine in male subjects, despite no statistically significant difference of the canine distance between genders. The size of upper anterior central incisors was accounted for approximately 47.9 % of the distance between two canines, when the percentages of central incisor width-to-canine distance were bilaterally combined (data not shown).

**Table 1:**
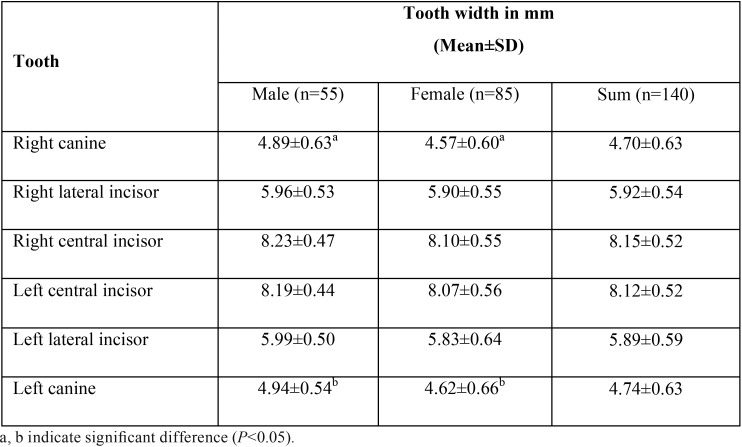
Average width of each upper anterior tooth.

**Figure 4:**
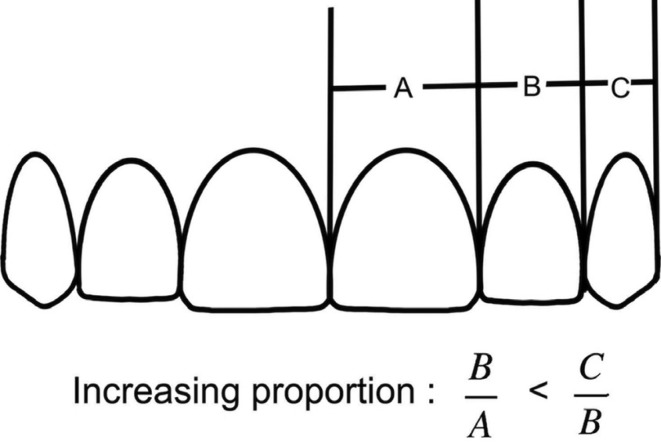
Schematic representation of an increasing proportion found in natural upper anterior teeth.

Next, relative ratio, referred here as the esthetic proportion, of lateral-to-central incisor and ratio of canine-to-lateral incisor width were calculated ( Table 2). We found a significant difference of the right canine-to-lateral incisor ratio between male and female as shown in  Table 2, possibly due to the discrepancy of the tooth size. A bilaterally combined ratio of canine-to-lateral incisor in female was therefore less than that derived from male subjects, although not statistically significant as shown in  Table 2. Considering the bilaterally combined esthetic proportion of lateral-to-central incisor and canine-to-lateral incisor reported from this study, natural upper anterior teeth display an increasing proportion: from a ratio of 0.72±0.06 to a ratio of 0.80±0.13 in a medio-distal direction, as depicted in Figure 4.

**Table 2:**
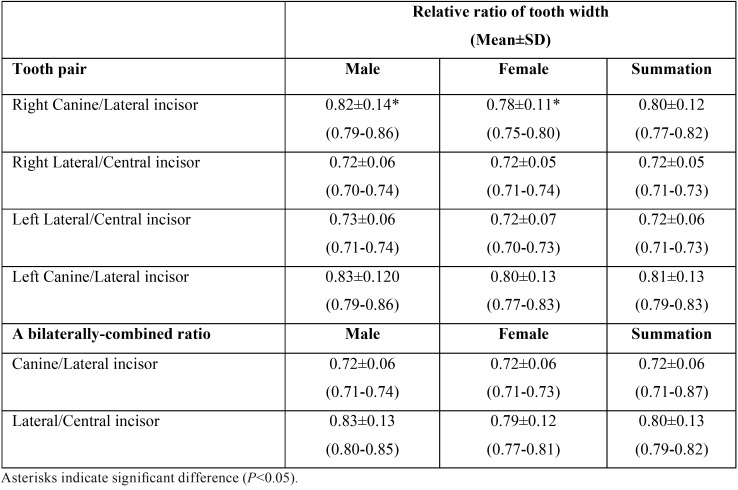
Relative ratio of tooth width (esthetic proportion) calculated from our studied population.

The esthetic proportion we reported was then compared to other 3 systems including golden proportion, 70% RED, and 80% RED. Three constant estimates i.e. 0.618, 0.7, and 0.8 indicate a golden proportion, 70% RED, and 80% RED, respectively. Remarkably, our reported esthetic proportions of the upper anterior natural teeth significantly differ from the golden proportion and 70% RED (*P*=0.001), clearly suggesting a limited validity of the golden proportion among our studied group. However, our calculated esthetic proportion of lateral incisor-to-central incisor (0.72±0.06 mm) was statistically different, when compared to 80%RED (*P*=0.001). Moreover, calculated golden percentage of each anterior tooth relative to canine-to-canine distance shown in  Table 3 also significantly differed from the proposed golden percentage, particularly for central incisors and canines.

**Table 3:**
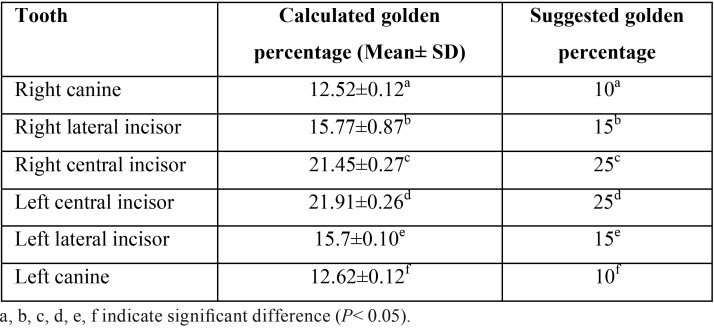
Comparison between a calculated and a suggested golden percentage.

According to our survey of the esthetic satisfaction of the variety of tooth proportions, we found a subtle difference of the satisfaction level between various images as shown in  Table 4. Interestingly, female individuals seemed to prefer an increasing proportion depicted (a score of 2.98±0.75) when compared to males in which 70% RED was highest rated (a score of 2.84±0.81), albeit no statistically significant difference (*P*=0.054). Notably, 70% RED was attributed to the most satisfactory level in our select population.

**Table 4:**
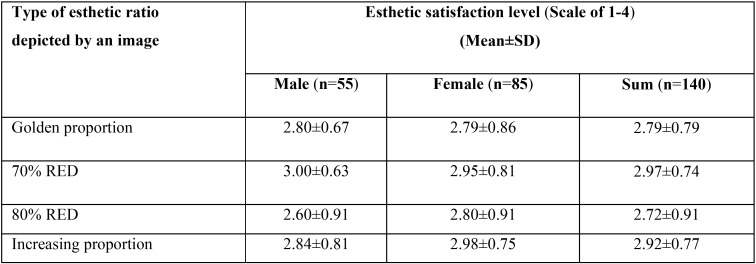
Esthetic satisfaction level of images illustrating 4 different esthetic ratios.

## Discussion

Dental esthetics is naturally contributed by the characteristics of upper anterior teeth, smiling components, and dentition (1-3). Anterior tooth restoration should therefore clinically take this into a crucial account. Fortunately, earlier clinical studies identifying the esthetic proportions of upper anterior teeth i.e. golden proportion, golden percentage, and RED have made clinical guidelines available (4-8). However, whether esthetic proportions reported from those studies can be used with Thai population warrants further study. Contrastingly, our pilot study has found that the proportion of natural upper anterior teeth is depicted as an increasing ratio from mesial tooth to it distal counterparts, inconsistent with previous studies in which the estimates of each maxillary anterior tooth were identical.

Measurement of tooth size can be performed by several methods, either direct or indirect method. Direct measurement intraorally or from plaster casts can be easily made, while an indirect measurement from the front-view photographs using a computer program similarly reported by previous studies (16,17) might be an alternative strategy since it truly represents the view of the teeth seen by other individuals (4). We adopted an indirect method suggested by Ward in 2001 (6) and also used a reference object of a known size to overcome the possibility of having a distorted photograph. Importantly, the calculated correlation coefficient greater than 0.8 has confirmed the reliability of our measurement method (data not shown). We hereby reported that the average width of upper anterior tooth was nearly bilaterally identical and the magnitude was not significantly different among genders, except the width of upper canine ( Table 1). Mean width of upper central and lateral incisors was 8.13 and 5.91 mm, respectively calculated from both genders. However, canine width was 4.91 mm in males while its was shown to be 4.60 mm in females. Our measured outcomes are comparable to those reported by Hasanreisoglu (17) with an exception of the mean width of central incisors. Hasanreisoglu reported a value of 8.6 mm; the race of study population might thus contribute to this discrepancy.

It is not yet conclusive whether gender has an impact on the width of upper anterior teeth. Nevertheless, our results have shown that there is no correlation between gender and mesio-distal width of upper central and lateral incisors. Interestingly, our results have suggested the existence of a correlation between canine width and gender. Recent studies have also demonstrated the clinical relationship between gender and the size of upper anterior teeth (14,17), particularly canine in which the size varies between males and females (17).

Esthetic proportions derived from upper natural anterior teeth in our study increased in a mesio-distal direction so called “increasing proportion” shown in Figure 3, similar to our initial findings. We found that a ratio of lateral-to-central incisor was 0.72, slightly greater than a ratio of 0.70 from our preliminary results. Interestingly, a ratio of canine-to-lateral incisor remained constant at 0.80. Preston suggested the variability of esthetic ratios derived from upper anterior teeth (10). While a ratio of canine-to-lateral incisor reported from his study was 0.84, a lateral-to-central incisor ratio was 0.66, far less than those estimate reported here from our finding. However, one study in 30 Indian subjects reported earlier that tooth width ratios vary, in a tooth height-dependent manner (18). Importantly, their study also found that canine to lateral incisor width ratio was slightly less than that of lateral incisor to central incisor width ratio, and the ratios were different from Ward’s report on RED. The discrepancy between study findings might be due to the utilization of distinct research tools. Most likely, different strategy of measurement for data acquisition and analysis, number of samples studied, ethnic groups, and selected individuals who had undergone an orthodontic treatment might significantly contribute to this data discrepancy.

Remarkably, esthetic proportions derived from this study are significantly different from the golden proportion of 0.618 (*P*<0.05). Our findings are in agreement with the notion that the occurrence of the golden proportion in natural teeth is not very likely. A clinical survey has indicated that only 17% of a lateral-to-central incisor ratio corresponds to the golden proportion, whereas there is no probability of finding a canine-to-lateral incisor of 0.618 (10). Even though the golden proportion was not frequently found in natural dentition, recent study suggested that tooth form corresponding to the golden proportion is more preferable by American dentists than other proportions (19). We calculated a golden percentage and found that the average tooth proportions calculated according to the concept of golden percentage are not closely similar to those of the golden percentage as demonstrated in  Table 3. Our findings are consistent with a recent report analyzing the width and the height of 384 subjects found that neither golden percentage nor RED proportion was followed (20).

In this study, we also aimed to survey the esthetic satisfaction of different smile patterns consisting of distinct tooth proportions in our studied population. Photographs depicting 4 different tooth sizes were shown to the participants and they were asked to score each photograph separately, corresponding to their personal preference and satisfaction. Remarkably, we found that a studied group preferred a photograph portraying 70% RED the most and the least satisfactory tooth form was a smile composed of 80% RED, shown in  Table 4. Proportion of the upper anterior teeth plays a significant role in contributing to an esthetic satisfaction level among dentists and the shape of upper central incisors is partially accountable for this (15,18). Essentially, our findings have highlighted that firstly, a patient satisfaction of dental esthetic profile is crucial for a clinical consideration potentially for achieving the most satisfactory treatment outcomes. Secondly, a level of personal appreciation of dental elements varies among patients. It is thus suggested that, under certain circumstance, dental professionals should therefore take this consideration into an account in order to surpass the patients’ expectation of the treatment outcome.

In summary, our results have indicated that the esthetic proportions derived from upper anterior teeth of indigenous Northeastern Thai population are in a range close to the ratios previously reported. However, golden proportion is not valid in our studied group. Notably, we suggest for the first time the existence of an increasing proportion in Thai population. It is therefore highly likely that tooth form differs among the heterogeneous population. A well-balanced form of natural upper anterior teeth clinically satisfies the patients’ desire of the esthetic outcome of the esthetic dental treatment. Therefore, the lack of esthetic, clinical considerations in dental treatment planning might be a tremendous hurdle to seizing a successful treatment. Our study provides an essential platform for the dental professionals to take the esthetic tooth ratios and a satisfaction of the patient into clinical consideration in order to bring about the most successful treatment outcome. However, additional studies of the tooth height proportional to the width, as well as a clinical survey of the patients’ self-satisfaction of their own tooth form and alignment, might provide a further clinical guideline and be a useful clinical tool for restoring anterior teeth to achieve a maximal patient satisfaction.
